# Rhodopsin Gene Expression Determines Rod Outer Segment Size and Rod Cell Resistance to a Dominant-Negative Neurodegeneration Mutant

**DOI:** 10.1371/journal.pone.0049889

**Published:** 2012-11-21

**Authors:** Brandee A. Price, Ivette M. Sandoval, Fung Chan, Ralph Nichols, Ramon Roman-Sanchez, Theodore G. Wensel, John H. Wilson

**Affiliations:** 1 Verna and Marrs McLean Department of Biochemistry and Molecular Biology, Baylor College of Medicine, Houston, Texas, United States of America; 2 Department of Molecular and Human Genetics, Baylor College of Medicine, Houston, Texas, United States of America; 3 Department of Ophthalmology, Baylor College of Medicine, Houston, Texas, United States of America; 4 Graduate Program in Cellular and Molecular Biology, Baylor College of Medicine, Houston, Texas, United States of America; University of Florida, United States of America

## Abstract

Two outstanding unknowns in the biology of photoreceptors are the molecular determinants of cell size, which is remarkably uniform among mammalian species, and the mechanisms of rod cell death associated with inherited neurodegenerative blinding diseases such as retinitis pigmentosa. We have addressed both questions by performing an *in vivo* titration with rhodopsin gene copies in genetically engineered mice that express only normal rhodopsin or an autosomal dominant allele, encoding rhodopsin with a disease-causing P23H substitution. The results reveal that the volume of the rod outer segment is proportional to rhodopsin gene expression; that P23H-rhodopsin, the most common rhodopsin gene disease allele, causes cell death via a dominant-negative mechanism; and that long term survival of rod cells carrying P23H-rhodopsin can be achieved by increasing the levels of wild type rhodopsin. These results point to promising directions in gene therapy for autosomal dominant neurodegenerative diseases caused by dominant-negative mutations.

## Introduction

Progressive neurodegenerative conditions represent a vast and growing medical problem in an aging population. Most such conditions appear to be multifactorial, with identification of risk factors in its very early stages, but even for those with known monogenic origins, the mechanisms by which deleterious mutations cause disease are poorly understood. The problem is especially difficult for diseases with autosomal dominant inheritance, as many and perhaps most of these are due to an unknown toxic mechanism of action of the affected protein. Autosomal dominant degeneration also presents a particularly daunting challenge for designing molecularly targeted treatments such as gene therapy, as simple gene replacement may not counteract the toxic effect.

Neurodegenerative diseases of the retina, and animal models of them, are an especially tractable system in which to begin to address this general problem. Mutations in the rhodopsin gene account for about 10% of all cases of retinitis pigmentosa (RP), a progressive hereditary blinding disorder than affects 1 in 4,000 people worldwide [1], [2]. Patients with RP often experience symptoms such as night blindness as adolescents, with ongoing loss of rod cell function leading ultimately to complete blindness. With only a few exceptions, the more than 150 rhodopsin mutations in the human population cause a dominant form of the disease [2] (http://www.sph.uth.tmc.edu/retnet/; http://www.hgmd.org/). The most common rhodopsin mutation in North America causes a proline-to-histidine change at codon 23 (P23H). Although P23H was the first RP mutation identified in human patients, its pathogenic mechanism is still not clear [3], [4]. Even the toxic site of action of P23H-rhodopsin remains controversial, with hypothesized mechanisms including endoplasmic reticulum (ER) stress, caused by the presence of mutant rhodopsin [5], [6], [7]; aberrant function of rod disk membranes, caused by incorporation of mutant rhodopsin [8], [9], [10]; and interference with synaptic function, caused by abnormal accumulation of mutant rhodopsin at the synaptic terminals [11].

As a first step, we reasoned that it would be important to distinguish between dominant-negative and gain-of-function mutations, and that determining the effects of increasing levels of wild type protein would allow this distinction to be made [12]. Two previous studies in mice suggest that P23H-rhodopsin may be a dominant-negative mutation based on improvement in the health of retinas carrying a P23H-rhodopsin transgene upon expression of additional wild type rhodopsin [5], [13]. However, studies over the last decade in cultured cells have variously suggested that P23H-rhodopsin is a toxic gain-of-function mutation [14], [15], a dominant-negative mutation [16], and a mixture of the two [17]. To resolve this issue, which is central to the pathogenic mechanism, we carried out a systematic study over a wide range of wild type rhodopsin expression levels. To vary wild type rhodopsin expression, we used mice carrying the NHRE transgene, which expresses human rhodopsin at about the same level as one endogenous mRho gene [18], in combination with a knockout allele [19] and normal mRho genes, to cover a range roughly equivalent to 0, 1, 2, 3, and 4 rhodopsin genes. By combining a P23H-rhodopsin transgene with these normal alleles, we were able to assess the effects of rhodopsin expression on rod cell structure and retinal neurodegeneration.

To identify the toxic site of action of P23H-rhodopsin, we also examined the distribution of an EGFP-tagged version of human P23H-rhodopsin expressed from the knockin allele, P23H-hRho-GFP [20]. We had previously found that P23H-rhodopsin-GFP is mostly mislocalized to the inner segment and outer nuclear layer and subject to degradation in mouse rods [20]. By contrast, its nonmutant counterpart, human rhodopsin-GFP, expressed from the hRhoG(H) knockin allele [21], is much more stable and is correctly targeted to the outer segment, the organelle in which wild type rhodopsin is almost exclusively localized [20]. Thus, P23H-rhodopsin-GFP makes a good marker for the fate of P23H-rhodopsin. In combination with different numbers of normal rhodopsin genes, we were able to measure changes in P23H-rhodopsin-GFP localization, providing insight into the site of toxic action of P23H-rhodopsin.

In the course of this work, we discovered a strikingly simple relationship between the level of rhodopsin expression and the size of the rod cells, specifically of the outer segments where it comprises the majority of the protein. These results provide new insights into a longstanding puzzle in cell biology: how sizes of cells and organelles are determined at the molecular level. These results are particularly applicable to cells containing primary cilia, as the rod outer segment is a modified primary cilium. They suggest that the expression level of a single protein, rhodopsin, governs the size of this organelle, and that this single parameter may explain both the high uniformity in outer segments among mammalian rods and the two order-of-magnitude variation in sizes between mammals and some cold blooded species, which accounts for the much faster response kinetics of the smaller mammalian rods.

## Results

### Rhodopsin Expression from the NHRE Transgene

**Figure 1:**
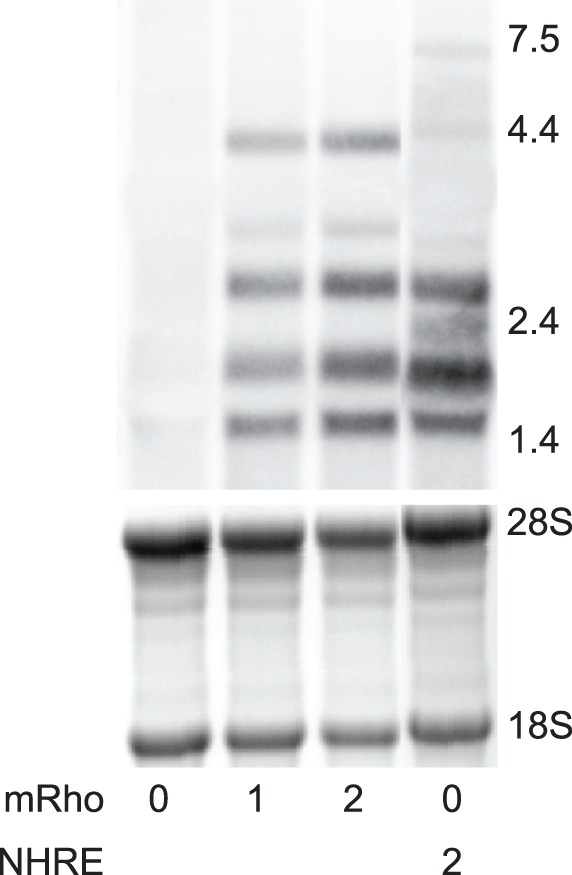
Rhodopsin expression in mice with various numbers of rhodopsin genes. (A) Northern blot analysis of rhodopsin mRNA from various mouse lines. Copy numbers of wild type rhodopsin and P23H-rhodopsin genes are indicated below each lane, where mRho 0 is mRho^−/−^, mRho 1 is mRho^+/−^, mRho 2 is mRho^+/+^, and mRho 0 NHRE 2 is mRho^−/−^NHRE^+/+^. Quantification of the 18S and 26S rRNAs bands from the agarose gels prior to transfer (shown as a negative below the Northerns) served as loading controls. Six retinas were used to prepare each mRNA sample. Northern images were obtained on a PhosphorImager. Sizes are indicated in kb.

To measure the levels of rhodopsin mRNA expression from the NHRE transgene, we compared NHRE^+/+^mRho^−/−^ mice with mRho^+/+^, mRho^+/−^, and mRho^−/−^ mice by Northern blot analysis at postnatal day 30 (P30) (Figure 1). Quantification of the bands relative to mRho^+/+^ mice showed that mRho^−/−^ mice did not express rhodopsin mRNA, whereas mRho^+/−^ mice made 53% as much mRNA as mRho^+/+^ mice, as expected, and NHRE^+/+^mRho^−/−^ mice synthesized 94% as much (Table 1). Using spectrophotometry, we also measured total rhodopsin protein levels in P30 retinas from mRho^+/−^, mRho^+/+^, and NHRE^+/+^mRho^−/−^ mice. Relative to wild type mice, mRho^+/−^ mice made 48% as much rhodopsin and NHRE^+/+^mRho^−/−^ mice made 90% as much (Table 1). These measurements showed that one NHRE transgene is roughly equivalent in expression to one endogenous mouse rhodopsin allele. The level of wild type mRNA we measured from the NHRE transgene was about half of that originally reported [18], possibly as a result of genetic or epigenetic changes during the 20-year breeding history of this line. For simplicity, we will refer to various combinations of mRho and NHRE alleles as containing 0, 1, 2, 3, or 4 copies of rhodopsin (Table 2).

**Table 1:** Rhodopsin expression from mRho and NHRE alleles.

Genotype	Copy number	mRNA(%)	Protein^a^(pmol)	Protein^a^(%)
mRho^−/−^	0	0	ND	ND
mRho^+/−^	1	53	205±25	48±6
mRho^+/+^	2	100	429±46	100±11
NHRE^+/+^mRho^−/−^	2	94	388±81	90±19

a For the spectrophotometric analysis of rhodopsin protein concentration, we performed a univariate ANOVA with a Least Squares Difference post-hoc analysis. For 1 versus 2 and 3 copies, *P* = 0.002 and 0.006, respectively. Two copies of mouse and two copies of human (NHRE) are not significantly different.

**Table 2:** Crosses to generate mice with different numbers of rhodopsin genes.

Crosses			Offspring	Copy Number
mRho^−/−^	X	mRho^−/−^	mRho^−/−^	0
mRho^−/−^	X	mRho^+/+^P23H^+/0^	mRho^+/−^	1
			mRho^+/−^P23H^+/0^	1+P23H
mRho^+/+^	X	mRho^+/+^P23H^+/0^	mRho^+/+^	2
			mRho^+/+^P23H^+/0^	2+P23H
mRho^+/+^NHRE^+/+^	X	mRho^+/+^P23H^+/0^	mRho^+/+^NHRE^+/0^	3
			mRho^+/+^NHRE^+/0^P23H^+/0^	3+P23H
mRho^+/+^NHRE^+/+^	X	mRho^+/+^NHRE^+/+^	mRho^+/+^NHRE^+/+^	4

### Effects of Rhodopsin Expression on Rod-cell Structure and Degeneration

**Figure 2:**
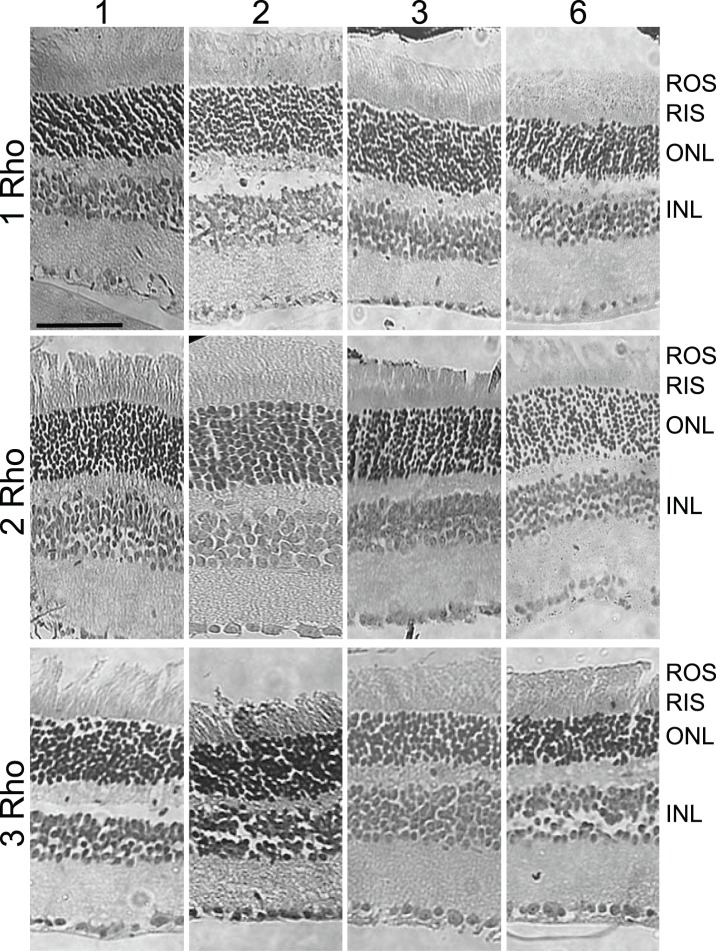
Hematoxylin and eosin stained images of retinal sections from mice with different numbers of wild type rhodopsin genes. Genotypes are indicated at left, where 1 Rho is mRho^+/−^, 2 Rho is mRho^+/+^, and 3 Rho is mRho^+/+^NHRE^+/0^. Ages in months are indicated at the top. Retinal layers are abbreviated at right, where ROS is rod outer segment, RIS is rod inner segment, ONL is outer nuclear layer, OPL is outer plexiform layer, INL is inner nuclear layer, and IPL is inner plexiform layer.

**Figure 3:**
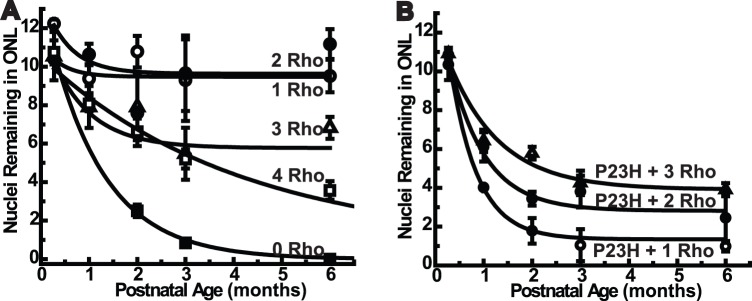
Measurements of retinal degeneration. (A) Counts of nuclei per column of nuclei in the outer nuclear layers (ONL) of retinas from mice with different numbers of wild type rhodopsin genes. Genotypes are abbreviated on the curves: 0 Rho is mRho^−/−^ (filled squares), 1 Rho is mRho^+/−^ (open circles), 2 Rho is mRho^+/+^ (filled circles), 3 Rho is mRho^+/+^NHRE^+/0^ (open triangles), and 4 Rho is mRho^+/+^NHRE^+/+^ (open squares). (B) Counts of nuclei per column of nuclei in the ONL of retinas from mice with the P23H-rhodpsin transgene. Genotypes are abbreviated on the curves: 1 Rho+P23H is mRho^+/−^P23H^+/0^ (open circles), 2 Rho+P23H is mRho^+/+^P23H^+/0^ (filled circles), and 3 Rho+P23H is mRho^+/+^NHRE^+/0^P23H (open triangles). We counted 60–100 columns of nuclei for multiple areas within each retina (3 eyes from 3 individual mice per genotype per timepoint) and averaged them for each time point. Error bars indicate standard error of the mean. Curves were fit to an exponential decay curve, allowing for a plateau value. Exponentials are from non-linear curve fitting using the Marquardt-Levenberg method in Origin, with weighting by 1/variance. The 3 Rho curve-fitting also included data from 3 mice at 8.5 months, which had 6.7 nuclei, and the 4 Rho curve-fitting included data from 1 mouse at 10 months, which had 1.5 nuclei (not shown).

To measure the effect of increasing the amounts of wild type rhodopsin on retinal structure and rod cell morphology, we generated mice that carried various numbers of rhodopsin genes. Analysis of H&E-stained retinal cross-sections revealed fairly normal overall retinal structure in mice with 1, 2, of 3 copies of rhodopsin at various ages (Figure 2). In wild type mice, after one postnatal month, the number of photoreceptor nuclei remained relatively constant for many months, as assessed by the number of nuclei per column of nuclei, which is a measure of thickness of the outer nuclear layer (Figure 3A). In contrast, mice suffering from progressive retinal degeneration typically undergo an exponential decline to zero nuclei [22], [23], [24]. We measured the rate of photoreceptor cell death in mice with 0, 1, 2, 3, and 4 copies of rhodopsin by counting the number of nuclei remaining in the outer nuclear layer at several time-points up to six months of age (Figure 3A). As shown previously, retinas in mice with no rhodopsin genes (mRho^−/−^) degenerated rapidly, whereas those with a single rhodopsin gene (mRho^+/−^) displayed a loss of nuclei that was not much different from wild type mice [19], [25]. Mice with 3 copies (NHRE^+/0^mRho^+/+^) exhibited an initial loss of nuclei from one to three postnatal months and then the decline appeared to slow greatly or stop, so that no loss was detected from month 3 to month 6. We confirmed the apparent plateau by showing that an average of 6.7 nuclei remained in retinas from three-copy mice that were 8.5 months old. Thus, after the initial decline, the remaining 60% of the original nuclei appeared to be stable. Mice with 4 copies (NHRE^+/+^mRho^+/+^) displayed a slow exponential decline that did not appear to reach a plateau, with about 30% of the initial number remaining at 6 months. The absence of a plateau was evident in the retina from a 10-month old four-copy mouse, which had an average of 1.5 remaining nuclei. Taken together with the effects of rhodopsin expression on cell size, described below, these results suggest that the cell death in 3-copy mice may result, not from some intrinsic toxicity of high rhodopsin levels, but rather from a secondary effect of cell crowding, which ceases once the crowding is relieved. The exponential decay without a plateau in 4-copy mice suggests that wild type rhodopsin is toxic at this level of expression.

### Effects of Rhodopsin Expression on Rod Outer Segment Morphology

**Figure 4:**
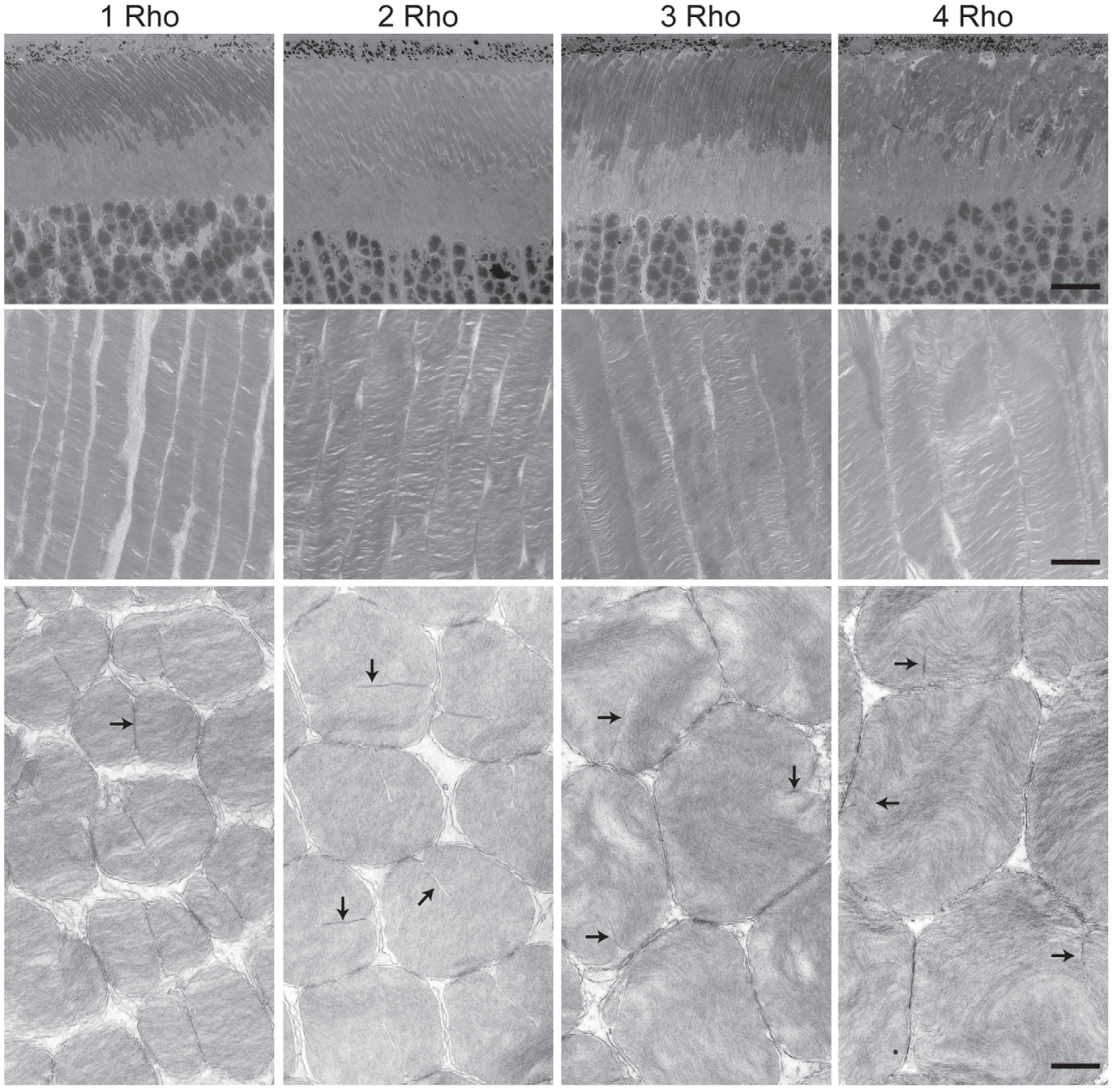
Electron micrographs of rod cells from 1-month old mice with different numbers of wild type rhodopsin genes. Genotypes are abbreviated at the top: 1 Rho is mRho^+/−^, 2 Rho is mRho^+/+^, 3 Rho is mRho^+/+^NHRE^+/0^, and 4 Rho is mRho^+/+^NHRE^+/+^. Arrows indicate incisures. The top row of images is at 700X magnification, the middle row is at 4400X, and the bottom row is at 12000X, with scale bars indicating 11 µm, 1.7 µm, and 0.6 µm, respectively.

**Figure 5:**
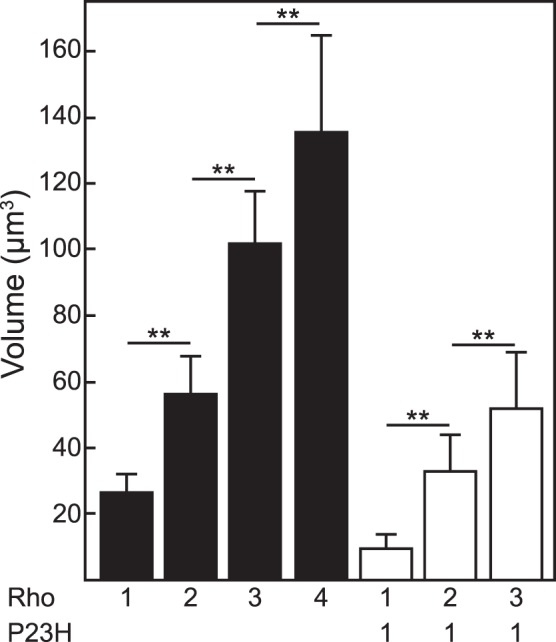
Volumes of rod outer segments from 1-month old mice. Volumes were calculated from measurements of width and length (Table 3), assuming that the rod outer segments are cylinders (V = πr^2^h). Genotypes are abbreviated below the figure: 1 Rho is mRho^+/−^, 2 Rho is mRho^+/+^, 3 Rho is mRho^+/+^NHRE^+/0^, 4 Rho is mRho^+/+^NHRE^+/+^, 1Rho+P23H is mRho^+/−^P23H^+/0^, 2 Rho+P23H is mRho^+/+^P23H^+/0^, and 3 Rho is mRho^+/+^NHRE^+/0^P23H^+/0^. Error bars represent the propagated error from the width and length measurements in Table 3, which were used in the calculation of volume. *P* values were determined for mice that differed by 1 copy of rhodopsin gene, using Student’s *t*-test. ** indicates *P*<0.001.

We used electron microscopy to analyze the morphology of rod outer segments in the retinas of mice with 1, 2, 3, or 4 copies of the rhodopsin gene at P30 (Figure 4). Overall, the structures of the rod outer segments in each of these mice were fairly normal, except for the obvious differences in size. The linear density of disks in the outer segments was the same for each copy number tested (Table 3), and incisures were visible in all (Figure 4, arrows). Measurements of rod outer segment parameters are given in Table 3. The calculated volumes of the outer segments, which assumed that rods were cylinders and thus approximated their true volumes, were proportional to rhodopsin copy number. This striking result implies that rhodopsin gene expression is sufficient to specify the size of the outer segment (Figure 5).

**Table 3:** Measurements of rod outer segments in P30 mice with increasing rhodopsin expression.

Copy Number	Genotype	Width^a^(µm)	Length^a^(µm)	Disks/µm^a^	Incisures^a^(µm)
1	mRho^+/−^	1.1±0.2	27.1±4.1	26.4±0.9	0.8±0.4
2	mRho^+/+^	1.5±0.2	32.1±4.6	25.2±1.2	0.7±0.2
3	mRho^+/+^NHRE^+/0^	1.8±0.2	40.1±4.2	24.3±0.9	0.6±0.2
4	mRho^+/+^NHRE^+/+^	2.2±0.4	35.8±3.9	25.3±0.9	0.5±0.3
1+P23H	mRho^+/−^P23H^+/0^	1.4±0.3	7.3±2.4		
2+P23H	mRho^+/+^P23H^+/0^	1.8±0.4	13.1±2.9		
3+P23H	mRho^+/+^NHRE^+/0^P23H	2.1±0.4	15.0±4.0		

a Boxer from EMAN [49] was used to measure the rod outer segment length, width, and incisure length. For width, we used longitudinal sections through the outer segments (see middle panels in Figures 4 and 7) and measured the widest section of the outer segment. Since most rods are cut at some angle relative to their long axis, this method helps to ensure an accurate measure of width. For each genotype, we made 20 measurements from multiple sections for each mouse and averaged the measures from 3 different mice. For length, we used low magnification images of longitudinal sections such as those shown in the top panels in Figures 4 and 7. We used the thickness of the rod outer segment layer, as measured along a line parallel to the primary orientation of the rod outer segments, as an indicator of the average length of rod outer segments. For each genotype, we made 20 measurements from multiple sections for each mouse and averaged the measures from 3 different mice. The linear density of disks along the length of the rod outer segment was measured by counting the number of disks per micron in images like those in the middle panels of Figure 4. For incisure lengths, we chose sections such as those in the bottom panels in Figure 4, and measured lengths in those sections where an incisure was readily visible. For 2 Rho and 3 Rho mice, we also looked for sections where the striations from mis-oriented disks were minimal; this was not possible for 1 Rho and 4 Rho mice, which displayed a more striated appearance than 2 Rho or 3 Rho mice. For each genotype, we made 10 measurements of incisure length for each mouse and averaged the measurements from 3 different mice. In all cases, the measurements were averaged and the standard deviation was determined.

### Effects of Rhodopsin Expression on P23H-induced Rod Cell Structure and Degeneration

**Figure 6:**
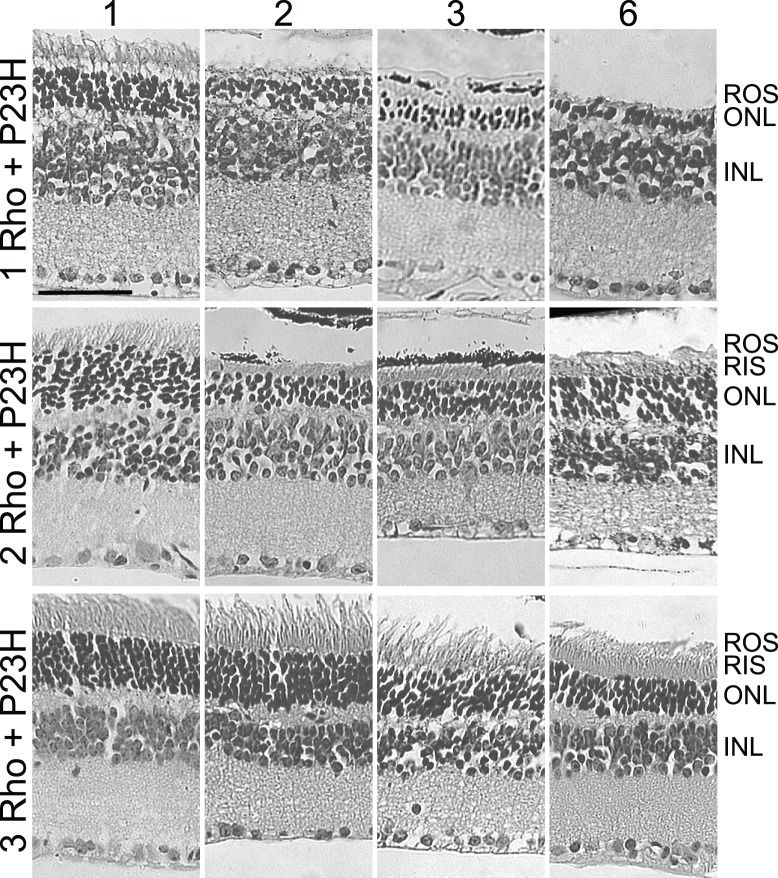
Hematoxylin and eosin stained images of retinal sections from mice with the P23H-rhodopsn transgene in combination with different numbers of wild type rhodopsin genes. Genotypes are indicated at left, where 1Rho+P23H is mRho^+/−^P23H^+/0^, 2 Rho+P23H is mRho^+/+^P23H^+/0^, and 3 Rho is mRho^+/+^NHRE^+/0^P23H^+/0^. Ages in months are indicated at the top. Retinal layers are abbreviated at right, where ROS is rod outer segment, RIS is rod inner segment, ONL is outer nuclear layer, OPL is outer plexiform layer, INL is inner nuclear layer, and IPL is inner plexiform layer.

To test the effects of rhodopsin levels on retinal degeneration induced by P23H-rhodopsin, we chose to use an extensively studied, slowly degenerating mouse line that contains a genomic mouse P23H-rhodopsin transgene [5], [26], [27]. This transgene had been previously shown to give rise to a steadily progressive retinal degeneration in the presence of two copies of the endogenous rhodopsin gene [5], with virtually no rod function remaining at 6 months, and an even faster degeneration in the presence of only one copy of the endogenous gene [5]. We bred the P23H-transgene onto backgrounds with 1, 2, or 3 copies of wild type rhodopsin. H&E staining revealed sparse rod outer segments in the presence of 1 copy of wild type rhodopsin, short rod outer segments with 2 copies, and longer rod outer segments with 3 copies (Figure 6). Although loss of nuclei occurs in all these mice, progression was slower with increasing expression of normal rhodopsin (Figure 3B). P23H mice with 3 copies of wild type rhodopsin retained about 40% of their photoreceptor nuclei at 6 months, and the degeneration at that age appeared to have dramatically slowed or disappeared. Mice with 3 wild type genes and the P23H transgene might be expected to display an ongoing retinal degeneration, instead of a plateau, as shown for mice with 4 wild type rhodopsin genes (Figure 3A). The P23H transgene, however, is transcribed only at about 50% the level of an endogenous rhodopsin gene [27] and about 80% of P23H-rhodopsin is degraded [5], [20]. Thus, mice with 3 wild type genes and the P23H transgene should have only slightly more total rhodopsin than mice with 3 wild type genes.

**Figure 7:**
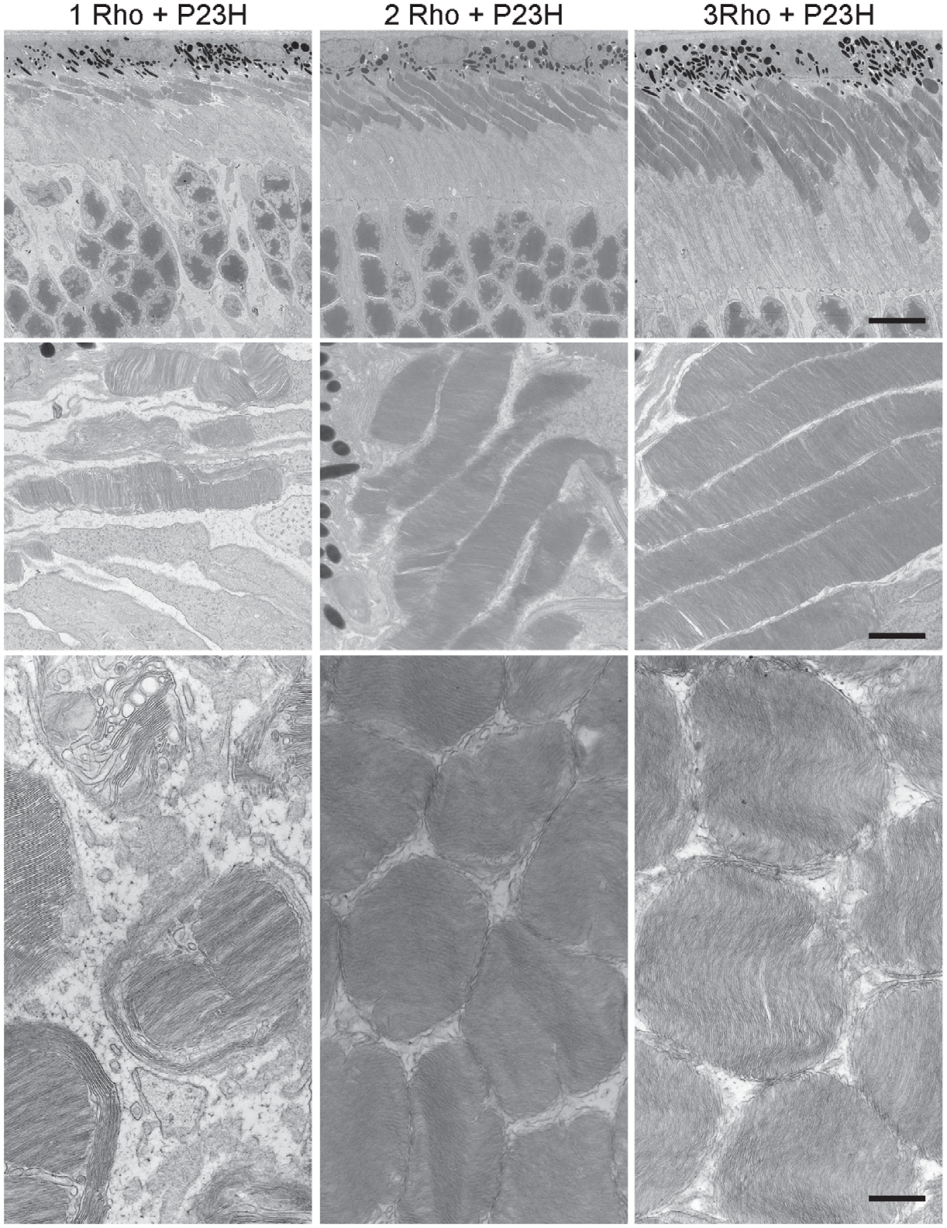
Electron micrographs of rod cells from 1-month old mice with the P23H transgene in combination with different numbers of wild type rhodopsin genes. Genotypes are abbreviated at the top: 1 Rho+P23H is mRho^+/−^P23H^+/0^, 2 Rho+P23H is mRho^+/+^P23H^+/0^, and 3 Rho+P23H is mRho^+/+^NHRE^+/0^P23H^+/0^. The top row of images is at 700X magnification, the middle row is at 4400X, and the bottom row is at 12000X, with scale bars indicating 11 µm, 1.7 µm, and 0.6 µm, respectively.

Electron microscopic examination of rod outer segments revealed rare photoreceptors in P23H mice with 1 wild type rhodopsin gene, and confirmed the improvement in ultrastructure with increasing copy number of normal rhodopsin (Figure 7). Measurements of rod cell dimensions (Table 3) also showed that the volume of the rod outer segment became more normal with increasing copies of rhodopsin (Figure 5). Thus, increasing the level of wild type rhodopsin conferred long-term resistance to the degenerative effects of the P23H mutant rhodopsin.

### Effects of Increased Rhodopsin Expression on Localization of Tagged P23H Rhodopsin

**Figure 8:**
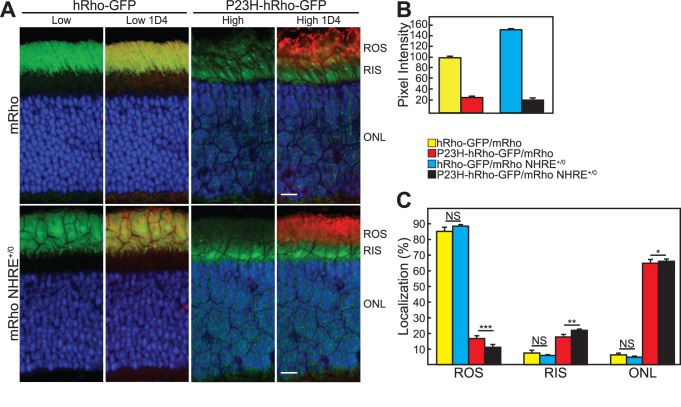
Distribution and quantification of GFP fluorescence in mice expressing hRho-GFP or P23H-hRho-GFP in the presence of 1 or 2 copies of wild type rhodopsin. (A) Retinas from age-matched hRho-GFP and P23H-hRho-GFP mice were sectioned and stained with DAPI. Sections were imaged using the heterozygous (hRho-GFP/mRho) sections to establish gain and laser power settings that gave limited numbers of saturated pixels in the region of the rod outer segments. These settings are referred to as “Low” and were also used to image retinas from hRho-GFP/mRho NHRE^+/0^ mice. Due to the lower amounts of P23H-hRho-GFP rhodopsin, retinas from P23H-hRho-GFP/mRho and P23H-hRho-GFP/mRho NHRE^+/0^ were imaged by increasing the laser power 5-fold, which is referred to as “High.” Immunostaining with 1D4 antibodies, shown in red, identifies the location of wild type rhodopsin. The scale bars are 10 µm. (B) Quantification of average GFP fluorescence intensity. The first and third images from the top and bottom rows in (A) were quantified using ImageJ and expressed relative to hRho-GFP/mRho retinas, which were defined as 100%. (C) Quantification of GFP localization. The amounts of GFP fluorescence the rod outer segments (ROS), rod inner segments (RIS), and outer nuclear layer (ONL) in images like those in the first and third columns of (A) were quantified using ImageJ. For the results displayed in (B) and (C), 5 different sections were examined from each of 4 hRho-GFP/mRho mice, each of 4 hRho-GFP/mRho NHRE^+/0^ mice, each of 8 P23H-hRho-GFP/mRho mice, and each of 8 P23H-hRho-GFP/mRho NHRE^+/0^ mice. Retinal sections were derived from various regions across the retina–excluding the periphery and the optic nerve. In all cases, error bars indicate standard deviations. We tested for statistical significance using a multivariate ANOVA with a Least Squares Difference post-hoc analysis. NS, not significant; *, *P*<0.05; **, *P*<0.001; ***, *P*<0.001.

To follow the localization of P23H-rhodopsin, we used two fluorescent knockin alleles–P23H-hRho-GFP and hRho-GFP–that are expressed at very low levels and do not cause retinal degeneration or interfere with rod cell function [20], [21]. The amounts and distributions of the fluorescent products of these alleles were measured in the presence of 1 copy of rhodopsin (mRho^+/−^) or 2 copies (mRho^+/−^ NHRE^+/0^). As shown in Figure 8A and B, the amount of hRho-GFP increased about 50% in the presence of 2 copies of rhodopsin, which was likely due to the increased size of the rod outer segments. The total amount of P23H-hRho-GFP, however, was only about 20% the level of hRho-GFP, indicative of its instability [20], and it remained approximately the same with increasing rhodopsin, presumably because most of it was mislocalized. The distribution of hRho-GFP was not significantly changed in retinas expressing 1 or 2 copies of wild type rhodopsin, with 85–90% in the outer segments (the fraction in the outer segment represents a lower limit due to saturation of some pixels) (Figure 8C). The distribution of P23H-hRho-GFP, however, changed significantly with increasing rhodopsin, with less detected in the outer segments and more in the inner segments and outer nuclear layer (Figure 8C). The lower expression of P23H-hRho-GFP in the outer segments, coupled with the increase in volume of the outer segments with increasing expression of wild type rhodopsin (Figure 5), means that the concentration of P23H-hRho-GFP in the disk membranes is substantially reduced in rod cells expressing 2 copies of wild type rhodopsin, relative to those expressing a single copy. The parallel improvement in rod cell morphology that accompanies the dramatic decrease in the concentration of P23H-hRho-GFP in disk membranes is consistent with the rod outer segment being the toxic site of action of P23H-hRho-GFP.

## Discussion

Four striking conclusions emerge from our *in vivo* titration of wild type rhodopsin in healthy and diseased retinas. First, there is a linear relationship between wild type rhodopsin expression and the size of rod outer segments over a wide range of expression levels. Previous comparisons of rod cell morphology in heterozygous rhodopsin nulls and wild type mice showed, with one exception [25], that the rod outer segments in mice with one rhodopsin gene are substantially smaller than those in wild type mice, in agreement with our measurements [19], [28], [29]. Additional studies have examined rod cell morphology in mice that over-express rhodopsin, using the “Bouse” transgene, which is a mouse gene with three amino acid substitutions that create an epitope from bovine rhodopsin [30]. Wild type mice carrying this transgene, which expresses the equivalent of one mouse rhodopsin gene, have larger outer segments, but their morphology is abnormal and the retinas rapidly degenerate [31]. Moreover, Bouse^+/0^ mRho^−/−^, Bouse^+/0^ mRho^+/−^, and Bouse^+/+^ mRho^+/+^ mice all display severe retinal degeneration at an early age [31], suggesting that the Bouse allele behaves like a mutant rhodopsin gene in mice [12], [31]. In contrast, the NHRE transgene used in these studies can support relatively normal outer segment development in all combinations with wild type and null mouse alleles [18], [24], [32]. The consistent increase in volume of rod outer with increasing rhodopsin expression indicates that the level of rhodopsin protein is sufficient to control the size of an organelle as complex as the rod outer segment, although it remains an open question how the amount of rhodopsin actually changes the physical structure of rod outer segments. For retinas expressing three rhodopsin genes, there is an inverse relationship between outer segment size and final number of rod cells, with cell death providing a mechanism for relieving crowding induced by increased outer segment size, a process akin to the elimination of excess neurons in the developing retina [33], [34].

Second, the pathophysiological mechanism by which the P23H mutation causes retinal degeneration has its molecular origins in a dominant-negative effect of the mutant protein, which can be largely overcome by increasing the levels of the wild type protein [12]. The parallel improvement in outer segment structure and rod cell survival with increasing copies of wild type rhodopsin, all in the presence of a single P23H transgene, indicates that P23H-rhodopsin interferes with the function of normal rhodopsin in a way that can be diluted out by extra wild type rhodopsin [3], [12], [13], a common genetic test to distinguish between dominant-negative and gain-of-function mutations [35], [36]. Transfection experiments in cultured cells have identified three possible dominant-negative interactions between P23H-rhodopsin and wild type rhodopsin: formation of intracellular protein aggregates containing both mutant and wild type rhodopsin [14]; impaired delivery of wild type rhodopsin to the plasma membrane in the presence of P23H-rhodopsin [16]; and enhanced proteasomal degradation of wild type rhodopsin in the presence of mutant rhodopsin [16]. While each of these potential dominant-negative effects could be diluted out by increased expression of wild type rhodopsin, none of them fit with our observation, as discussed below, that the critical pathogenic event occurs in the rod outer segment, a site of interaction that could not have been discovered by experiments in cultured cells. A likely site of dominant-negative interaction in the rod outer segment has been identified in a recent study, which demonstrated that P23H-rhodopsin destabilizes rod photoreceptor disk membranes in mice and frogs in a way that depends on the concentration of P23H-rhodopsin in disk membranes [37]. The increasing size of disks with increasing expression of wild type rhodopsin observed in our studies would be expected to lower the concentration of P23H-rhodopsin in the disk membranes, thereby reducing its deleterious effects.

Third, the correlation between improved long-term rod cell survival in P23H mice and the decreased concentration of P23H-rhodopsin in rod outer segments with increasing wild type rhodopsin suggests that the toxic site of action of P23H-rhodopsin is in the rod outer segment. Previously, the rod outer segment has been suggested as the toxic site, based on the abnormal appearance of the outer segments in mice expressing P23H-rhodopsin [5], [8], [10]. Indeed, the gnarled outer segments with misoriented disks, readily visible in mice expressing the P23H-rhodopsin transgene along with 1 wild type copy of rhodopsin, largely disappear in mice expressing 2 or 3 copies (Figure 7). Because most GPCRs appear to traffic to the plasma membrane as homo- and heterodimers [38], we had expected that increased expression of wild type rhodopsin would, through its ability to heterodimerize, help to chaperone P23H-rhodopsin through the ER and Golgi to the rod outer segment, leading to less P23H-rhodopsin in the inner segments and outer nuclear layer and more in the rod outer segments with increasing expression of wild type rhodopsin. The opposite effects of wild type expression on the cellular distribution of P23H-rhodopsin observed in our experiments (Figure 8C) suggest that wild type rhodopsin heterodimerizes with P23H-rhodopsin weakly, if at all. The improvement in retinal health associated with loss of P23H-rhodopsin from the outer segments and gain in the inner segments and outer nuclear layer, where mislocalized P23H-rhodopsin is associated with the ER [5], make it unlikely that ER stress, due to excessive levels of P23H-rhodopsin trapped in the ER, is the cause of cell death [6], [7]. Additionally, if P23H-rhodopsin triggered cell toxicity were due to ER stress [6], [7], which is considered a gain-of-function mutation [3], rod cell structure and survival would not be expected to improve with increasing expression of wild type rhodopsin.

Fourth, increasing wild type rhodopsin levels up to three times the amount expressed in a retina heterozygous for a mutant P23H-rhodopsin allele protects the retina from degeneration. Thus, treatments that elevate rhodopsin levels, like AAV-mediated gene transduction [13], hold promise for ameliorating diseases caused by dominant-negative mutants of rhodopsin, provided the levels induced are carefully calibrated so as not to exceed those tolerated by the rod cells. It is unclear how many of the more than 150 rhodopsin mutations that cause RP in the human population are dominant-negative, and thus amenable to this approach, but we have demonstrated a general strategy for identifying them. Our studies also define the safe range of total rhodopsin, which has been a matter of some controversy [18], [30], [31], as between three and four gene-equivalents of rhodopsin. We do not know whether expression of extra rhodopsin will alter the dimensions of the rod outer segments in adults, since the extra rhodopsin in our studies was present during the early development of the rod cells, but this may be a critical question for therapeutic strategies that include delivery of a rhodopsin gene. Careful regulation of added rhodopsin is required, for example, for a variety of mutation-independent strategies–so-called kill-and-replace strategies–involving shRNA [39], ribozymes [40], [41], [42], or endonucleases [20], [43], which are designed to knock out or knock down both the mutant and wild type rhodopsin genes and restore wild type function by providing a treatment-resistant version [44], [45].

## Materials and Methods

### Ethics Statement

All animal procedures were carried out according to protocols approved by the Baylor College of Medicine Institutional Animal Care and Use Committee (IACUC), and in accordance with the Statement for the Use of Animals in Ophthalmic and Visual Research of the Association for Research in Vision and Ophthalmology.

### Animal Care and Genotyping

Mouse were housed under controlled conditions and monitored continually by laboratory personnel and by the professional staff and veterinarians of the Center for Comparative Medicine at Baylor College of Medicine in accordance with approved protocols from the IACUC. The experiments described here did not cause pain or suffering; however, the mouse were continually monitored for signs of pain or distress, and were treated or euthanized, as appropriate under approved protocols from the IACUC. Ultimately, mice were euthanized according to approved protocols from the IACUC. The mRho null mice, which were generated on an R1 background (129/SvJ × 129/Sv) [19], were obtained from Dr. Jane Farrar in combination with the heterozygous NHRE transgene, which was generated on a B6D2F background [18]. We separated the mRho null allele from the NHRE allele by outcrossing to C57Bl/6 mice to generate two homozygous lines: mRho^−/−^ and NHRE^+/+^mRho^+/+^. The P23H-rhodopsin transgenic mice, which contain 2–5 copies of a genomic mouse transgene carrying 5 mutations that lead to 3 closely linked amino acid changes (V20G, P23H, and P27L), were obtained from Dr. Wolfgang Baehr. The P23H-rhodopsin transgenic mice were generated on a C57BL/6 × SJL background and then backcrossed to C57Bl/6 mice [27]. Mice with different numbers of wild type rhodopsin genes in the presence or absence of the P23H-rhodopsin transgene were generated as summarized in SI Table 1. The hrhoG(H) knockin mouse line [21], which we will hereafter refer to as the hRho-GFP knockin mouse line, expresses human rhodopsin-GFP (hRho-GFP). It was backcrossed to C57BL/6 mice for more than 10 generations. The P23H-hRho-GFP knockin mouse line [20], which expresses human P23H-rhodopsin fused at its C-terminus to GFP, was backcrossed to C57Bl/6 mice for 6 generations at the time of these experiments. We determined mouse genotypes by PCR analysis of tail DNA. To assay for the P23H-rhodopsin transgene, we used primers W75 (5'-TGAGGCCACCAGACTGACATGGGGAGGAATTCCCAGA) and W11 (5'-GCCTGTGATCACAGCACTTGAGAGGCTGGG), as previously described [27]. We then performed a nested PCR using primers W11 and mRho exon 1a (5'-GAACGGCACAGAGG GCC). The nested PCR products were digested with NcoI (New England Biolabs, Ipswich, MA), which digests the wild type allele into two products (800 bp and 200 bp), but fails to cut the P23H-rhodopsin transgene [27]. The products were subjected to electrophoresis on a 0.8% agarose gel and samples containing the 1000-bp band were identified as P23H. To assay for the rhodopsin knockout (mRho^−^) allele, we used primers NeoR (5'-TTCAAGCCCAAGCTTTCGCG), exon IIF (5'-AGGTTAGAGCTGGAGGACTG) and exon IIR (5'-TAAGACTGATTGGACCATTC) [19]. The products were subjected to electrophoresis on a 0.8% agarose gel; a 200-bp band indicated the rhodopsin knockout allele. To assay for the human rhodopsin allele, we used primers hRho -412 to -392F (5'-GAGCTCCTCTGGGC AGGGCTG) and hRho -18 to -37R (5'-GGTCCCCTAACTTCTGCATG) [18]. The products were subjected to electrophoresis on a 0.8% agarose gel; a 400-bp band indicated the human rhodopsin allele.

### Northern Blotting

RNA was isolated from retinas of P30 mice obtained in the morning, as previously described [21], homogenized in TRI Reagent (Ambion, Life Technologies, Grand Island, NY) by using RNase-free plastic tubes and pestles. Genomic DNA was sheared by at least ten passes through a 20 gauge needle, 1-bromo-3-chloro-propane (10% by volume) was added, and the sample was shaken vigorously and then centrifuged at 12,000×*g* for 15 min at 4°C. RNA was extracted from the aqueous phase by using an RNeasy mini-kit (Qiagen, Valencia, CA), according to the manufacturer’s recommendations. Samples of total RNA were subjected to electrophoresis on 1% agarose denaturing formaldehyde gel (0.22 M formaldehyde, 20 mM MOPS buffer, pH 7.0, 5 mM sodium acetate, 1 mM EDTA), transferred to a Nylon membrane (Hybond-N+, Amersham Biosciences, GE Healthcare, Piscataway, NJ), and probed with an equal mixture of the coding regions of human and mouse rhodopsin cDNA, which was labeled by random priming in the presence of ^32^P-dCTP (DECAprime II Kit, Ambion, Life Technologies, Grand Island, NY). To normalize for loading, the blot was compared to the ribosomal subunit RNAs in the agarose gel prior to transfer. Samples were quantified by scanning the storage phosphor screen with Typhoon TRIO Variable Mode Imager (GE Healthcare, Piscataway, NJ) and analyzed with ImageJ [46].

### Protein Quantification

Total rhodopsin levels were measured by difference spectrophotometry. Mice were dark-adapted overnight and euthanized by cervical dislocation. Under dim red light conditions, whole eyes were immediately extracted in the morning following overnight dark-adaptation, frozen in liquid nitrogen and stored at −80°C until ready to use. For spectrophotometry, each single eye was homogenized with rotor and pestle in 240 µL of ROS buffer [1 mM MOPS, pH 7.4; 3 mM NaCl; 6 mM KCl; 0.2 mM MgCl_2_; 0.1 mM dithiothreitol (DTT); 0.02 mM phenylmethylsulfonyl fluoride (PMSF)] supplemented with 1.5% W/V LDAO (lauryldodecylamineoxide), 50 mM hydroxylamine and 1X proteinase inhibitor cocktail (Cat#11460400, Roche Applied Science, Indianapolis, IN). The samples were spun down at 200 × *g* and supernatant used directly for spectrophotometry. Absorbance spectra were recorded at room temperature using an Olis-modified SLM-Aminco DW-2000 dual-beam instrument (Olis, Bogart, GA). Rhodopsin concentration was calculated by difference absorbance at 500 nm using and extinction coefficient of 42,700 M^−1^cm^−1^ [47].

### Histology and Nuclear Counts

For hematoxylin and eosin staining, we fixed eyes in 10% Neutral Buffered Formalin (BDH, VWR, Radnor, PA) overnight at RT on a nutator. The fixed eyes were stored in 70% EtOH until processed at the Baylor College of Medicine Breast Cancer Histology Core and paraffin embedded (Shandon Histocentre 2, Thermo Scientific, Kalamazoo, MI). The paraffin blocks were immersed in 50% glycerol for several days prior to sectioning on a Leica RM2155 microtome (Leica Microsystems, Buffalo Grove, IL). The 5- to 6-µm sections were stained with hematoxylin and eosin. Briefly, sections were deparaffinized with three 5-minute washes of xylene, and rehydrated with two 5-minute washes of 100% EtOH, and a 3-minute wash each of decreasing gradient series of ethanol solutions. The slides were rinsed with distilled water and immersed in hematoxylin (VWR, West Chester, PA) for 2 minutes. Following hematoxylin, the slides were rinsed with distilled water and immersed in 0.25% Acid EtOH (70%) 5 times. The slides were rinsed with distilled water and then dipped in 10% Lithium Carbonate 5 times. Again, the slides were rinsed with distilled water and then dipped in 70% EtOH 10 times. The slides were immersed in 1% Eosin (VWR, West Chester, PA) for 2 minutes and then dehydrated by increasing concentrations of EtOH (95%–100%) twice each for 3 minutes. The staining was completed with 3 immersions in xylene for 2 minutes. The slides were coverslipped using Permount (Fisher Scientific, Kalamazoo, MI). We counted 60–100 columns of nuclei for multiple areas within each retina (3 eyes from 3 individual mice per genotype per timepoint) and averaged them for each time point. For nuclear counts, we used eye sections from the middle of the eye, where the plane of section is orthogonal to the retina. For the P8 and P14 time points, we fixed eyes in 4% paraformaldehyde in PBS at room temperature for 1 hour. After fixation, we soaked eyes in 30% sucrose in PBS overnight at 4°C until the eyes sank, and froze them in 100% Tissue-Tek OCT (Sakura Finetek, Torrance, CA) on dry ice. We cut 10- to 20-µm thick sections with a Microm HM500 microtome (Microm Instruments, Heidelberg, Germany), air-dried and kept them at −20°C until use. We stained eye sections using DAPI (Vector Labs, Burlingame, CA) in Vectashield (Vector Labs, Burlingame, CA). We captured images on a Leica TCI SP5 confocal microscope (Leica Microsystems, Buffalo Grove, IL) from several different locations in the retina, excluding areas around the optic nerve and the periphery. We measured ONL thickness using ImageJ [46].

### Immunostaining

Ten- to 20-µm sections were cut with a Microm HM500 microtome (Microm Instruments, Heidelberg, Germany), air-dried, and kept at −20°C until use. Eye sections were post-fixed in 50% methanol:50% acetone for 10 minutes at room temperature and then washed three times in 1X PBS. We blocked the sections overnight at 4°C in blocking buffer (10% normal donkey serum, 1% BSA, 0.5% Triton X-100) and then washed three times in 1X PBS. We incubated the eye sections with a 1∶500 dilution of 1D4 antibody overnight at 4°C in 3% normal donkey serum, 1% BSA, 0.5% Triton X-100 and then washed them three times in 1X PBS. The sections were stained with 1∶500 dilution of Alexa Fluor 594 goat anti-mouse secondary antibody (Life Technologies, Carlsbad, CA) and with 10 µg/mL DAPI (Roche Applied Science, Indianapolis, IN) for two hours at room temperature in 3% normal donkey serum, 1% BSA, 0.5% Triton X-100. We washed the sections three times in 1X PBS and stored them at −20°C until imaging on an Olympus FV1000 confocal microscope (Center Valley, PA), using a 60X objective.

### Electron Microscopy

Mice were euthanized in the morning and eyeballs were enucleated. The cornea and lens were immediately removed and the eyecup was fixed in Karnovsky buffer (2% paraformaldehyde, 2.5% glutaraldehyde, 0.1 M cacodylate buffer) (Electron Microscopy Sciences, Hatfield, PA) overnight at 4°C. Next morning, the tissue was washed twice with 0.01 M PIPES and postfixed in PIPES-buffered osmium tetroxide, pH 7.2, for 1 hr at room temperature (Electron Microscopy Sciences, Hatfield, PA). The tissue was rinsed in several changes of distilled water and dehydrated through a graded series of ethanol solutions. The dehydrated tissue was infiltrated in two 1.5-hr changes of propylene oxide followed by an overnight incubation in a 1∶1 mixture of propylene oxide and Spurr’s resin. The infiltrated tissue was incubated in pure resin for 1.5 hr and transferred to fresh resin in block molds. One-micron thick and ultra thin 60-nm thick sections were obtained. One-micron sections were stained with toluidine blue. Ultra thin sections were cut and mounted on mesh copper grids and stained with 2% uranyl acetate and Reynolds lead citrate. Grids were examined on a Jeol 100C Temscan electron microscope (Jeol, Peabody, MA) [48]. Electron micrographs were made on Kodak 4489 EM film (Kodak, Rochester, NY) and were digitized by scanning to a Dell computer (Dell, Round Rock, TX) with a Nikon Super Coolscan 9000 ED (Nikon Inc., Melville, NY). Boxer from EMAN [49] was used to measure the rod outer segment length, width, and incisure length. We measured three different mice per genotype. For length and width, we made 20 measurements per mouse; for incisures, we made 10 measurements per mouse. The measurements were averaged and the standard deviation was determined.

### Statistical Analysis

Univariate or multivariate ANOVA using SPSS grad pack (IBM, Armonk, NY) was used for statistical analysis. For all analyses, we measured three mice per genotype. For the spectrophotometric analysis of rhodopsin protein concentration, we performed a univariate ANOVA with a Least Squares Difference post- hRho/mRho, hRho/NHRE/mRho, P23H/mRho, and P23H/NHRE/mRho mice (Figure 8), we conducted a univariate ANOVA. For analyzing the differences in GFP-tagged rhodopsin localization (Figure 8), we measured five different sections per mouse and performed a multivariate ANOVA with a Least Squares Difference post-hoc analysis. For rod outer segment volumes, we compared mice that differed by 1 copy of the rhodopsin gene, using Student’s *t*-test.
